# Enhancing delivery of osteoarthritis care in the general practice consultation: evaluation of a behaviour change intervention

**DOI:** 10.1186/s12875-018-0715-8

**Published:** 2018-02-06

**Authors:** Mark Porcheret, Chris Main, Peter Croft, Krysia Dziedzic

**Affiliations:** Arthritis Research UK Primary Care Centre, Research Institute for Primary Care & Health Sciences, Keele University, Staffordshire, ST5 5BG UK

**Keywords:** Osteoarthritis, Primary care, Consultations, Behaviour change, Implementation, Simulated patients, Evaluation, Video-recording

## Abstract

**Background:**

Professionally-focussed behaviour change intervention (BCI) workshops were utilised in the Management of OsteoArthritis in Consultations (MOSAICS) trial investigating the feasibility of implementing the National Institute for Health and Care Excellence (NICE) Osteoarthritis (OA) Guideline in general practice. The workshops aimed to implement the general practitioner (GP) component of the trial intervention: an enhanced consultation for patients presenting with possible OA. This study presents an evaluation of the BCI workshops on GP competency in conducting these enhanced consultations.

**Methods:**

A before-and-after evaluation of the workshops, delivered to GPs participating in the intervention arm of the MOSAICS trial, using video-recorded GP consultations with simulated OA patients. GPs attended four workshops, which had been developed using an implementation framework. Videos were undertaken at three time-points (before workshops and at one- and five-months after) and were assessed by independent observers, blinded to time points, for GP competency in undertaking 14 predetermined consultation tasks.

**Results:**

Videos of 15 GPs were assessed. GP competency increased from a median of seven consultation tasks undertaken by each GP at baseline to 11 at both time-points after the workshops. Specific tasks which were undertaken more frequently after the workshops related to explaining that OA is treatable and not inevitably progressive, eliciting and addressing patient expectations of the consultation, and providing written OA information. However, the use of the word “osteoarthritis” in giving the diagnosis of OA was not enhanced by the workshops.

**Conclusions:**

BCI workshops can enhance GP competency in undertaking consultations for OA. Further initiatives to implement the NICE OA Guideline and enhance the care of people with OA in primary care can be informed by the content and delivery of the workshops evaluated in this study.

**Electronic supplementary material:**

The online version of this article (10.1186/s12875-018-0715-8) contains supplementary material, which is available to authorized users.

## Background

Osteoarthritis (OA) is a highly prevalent long-term condition in older adults for which extensive recommendations on assessment and treatment have been published [1–5]. It is predominantly managed in primary care, notably in the UK in general practice, but surveys of care, and interviews with patients and professionals, indicate that management of OA in general practice is suboptimal [6–8].

One aspect of suboptimal OA care is the conduct by general practitioners (GPs) of consultations for older patients presenting with peripheral joint pain, those likely to have OA. Evidence suggests that elements of the consultation could be improved, including: making the diagnosis of OA clinically; providing accurate information about the condition and on prognosis; promoting and supporting the use of non-pharmacological treatments; and adopting a patient-centred approach [9–11].

In the UK the National Institute for Health and Care Excellence (NICE) recommends that: OA should be diagnosed clinically; a holistic assessment should be undertaken; OA self-management should be supported; core treatments should be information, advice on activity and exercise and, if relevant, interventions to achieve weight loss; non-pharmacological and pharmacological treatments, and referral for consideration of joint should be used as additional treatment options [3, 12]. To implement these recommendations in UK general practice they would need to be acted on by GPs – to whom the majority of people with possible OA first present. Given current evidence of suboptimal GP consultations for OA, implementation of NICE OA guidance would need to include activities to enhance GP OA consultations.

This approach was taken in the Management of OsteoArthritis in Consultations (MOSAICS) trial, a cluster randomised trial to investigate the impact of implementing the 2008 NICE OA Guideline [13]. The trial intervention was a “model OA consultation” delivered by intervention arm practices to patients aged 45 years and over presenting to their GP with peripheral joint pain. This consisted of an OA Guidebook, an “enhanced initial OA consultation” with the GP, and a nurse-led OA clinic. A behaviour change intervention (BCI) was developed, and delivered as a series of BCI workshops, to implement the delivery of the “enhanced initial OA consultation” by GPs in intervention arm practices [14].

When evaluating the impact of behaviour change interventions, direct measurement of the intended behaviour, such as GP performance in day-to-day clinical practice, is recommended wherever possible, but indirect proxy measures are available when this is not practical [15]. Such proxy measures include the use of patient report, analysis of medical record entries, and the use of video-recorded consultations with simulated patients to measure competency – what someone is capable of doing in “controlled representations of professional practice” [16]. In the context of the MOSAICS trial it was not practical to directly measure GP performance in delivering the “enhanced initial OA consultation” – due to the logistics of recording such consultations in day-to-day practice – and a proxy measure was chosen.

This study aimed to evaluate the success of BCI workshops to implement delivery by GPs of the “enhanced initial OA consultation” by measuring workshop impact on GP competency to conduct these consultations using video-recorded consultations with simulated patients.

## Methods

### Design

A before-and-after evaluation of BCI workshops (for simplicity henceforth referred to as “the workshops”) was conducted using paired data on video-recorded consultations between GPs and simulated patients presenting with joint pain. GPs were video-recorded at their practices at three time-points: baseline before the workshops, and 1 month and 5 months after the workshops.

### Setting and participants

The study took place in four UK general practices which were participating in the intervention arm of the MOSAICS trial. All GPs working in these practices were invited to attend the workshops on OA management and delivery of the “enhanced initial OA consultation”. Eligible GPs for this study were those working in one of the practices and who had a video-recorded consultation at each time-point.

### The workshops

The development of the content and style of the workshops has been described in detail elsewhere [14]. In brief they were developed using an implementation of change model [17], presented the GPs with a “concrete proposal for change” [17], addressed “determinants of change” identified using the Theoretical Domains Framework [18], and incorporated systematically selected behaviour change techniques [19]. They consisted of four workshops, which used a mixture of didactic and interactive sessions, were learner-centred and facilitated by local opinion leaders, principally addressed identified knowledge and skills gaps relevant to delivery of OA consultations, and were mainly delivered at the GP practices. Full details are given in Additional file 1 using the Guideline for Reporting Evidence-based practice Educational interventions and Teaching (GREET) checklist [20].

### Video-recorded consultation data capture

Five simulators, who each portrayed a simulated patient with a different “story”, were recruited and trained for the study. At each time-point a research nurse and simulated patient attended the practice and GPs were invited, during an organised break in morning surgery, to undertake a simulated consultation (one at each time-point). At baseline (before the workshops) GPs were instructed to undertake the consultation as they would normally do, at the two later time-points (after the workshops) they were asked to undertake the consultation as promoted in the workshops. At each time-point the simulated patient, and so the scenario he or she presented, was new to the GPs, so providing a proxy for a new patient first presenting with a new problem (see Additional file 2).

### Assessment of video-recorded consultations

Videos were assessed for the presence of 14 predetermined OA consultation tasks. The tasks were the elements of the “enhanced initial OA consultation” which had been identified from a consensus study [21] as key elements of an initial consultation between a GP and an older adult presenting with peripheral joint pain (Table 1).

**Table 1:** Osteoarthritis (OA) consultation tasks assessed in the video-recorded consultations between GPs and simulated patients

Giving the diagnosis	
1.1	The GP elicits the patient’s ideas or worries or concerns about what they think is the matter with them, or the cause of their problem
1.2	The GP tells the patient the problem is due to OA, the word osteoarthritis needs to be used
Explaining the diagnosis	
2.1	The GP elicits what the patient knows or understands about OA, the word osteoarthritis needs to be used
2.2	The GP tells the patient that OA does not always / inevitably get worse, the word osteoarthritis does NOT need to be used
2.3	The GP tells the patient that OA is treatable: that there are things which can be done to help, the word osteoarthritis does NOT need to be used
Addressing expectations	
3.1	The GP elicits the specific expectation(s) the patient has of the GP about the problem
3.2	The GP responds to the patient’s specific expectations (as noted at 3.1)
Providing analgesia	
4.1	The GP elicits what the patient has tried or is trying for the problem
4.2	The GP advises about, or prescribes for, pain relief
Promoting self-management	
5.1	The GP elicits what the patient has tried or is trying for the problem, other than for the pain
5.2	The GP tells the patient that exercise(s) or physical activity is beneficial for patients with OA or for the patient’s problem
5.3	The GP tells the patient that losing weight, or not being overweight, is beneficial for patients with OA or for the patient’s problem
Promoting self-management support	
6.1	The GP offers, or gives, the patient general written information on OA
6.2	The GP offers, or gives, the patient an appointment with a practice nurse to help with OA

Videos were assessed by four trained assessors (GPs who were all independent of the MOSAICS trial), using a rating tool whose validity, and reliability in use, had been established (for details of assessor training, and validity and reliability testing see Additional file 3). Assessors were instructed to decide for each video and for each task whether the task had been undertaken or not, i.e. a binary assessment of “task present” or “task not present”. Each assessor was randomly allocated a set of trial GPs’ videos, (those of three or four GPs) and they assessed in random order all the videos of each of their allocated GPs, blinded to time-point.

### Analysis

Duration of videotaped consultations was calculated in minutes, and paired *t* tests were used to compare duration at baseline with that at one- and five-months.

We sought to measure both the competence of an individual GP in conducting an entire consultation, and the extent to which an individual task was undertaken by all the GPs at a given time-point. Two summary measures were therefore defined:

1. GP competency score: the number of tasks assessed as present in each video.
2. Task delivery score: the number of videos at a given time-point in which the task was assessed as present.

GP competency score was determined for each GP at each time-point. For each time-point median, interquartile range and range of competency scores across all GPs were calculated. Wilcoxon matched pairs signed-rank sum test [22] was used to compare median GP competency score at baseline with that at one- and five-months.

Task delivery score was calculated for each consultation task at each time-point and McNemar test with continuity correction (2-sided) [23] was used to compare scores at baseline with those at one- and five-months.

## Results

**Fig. 1:**
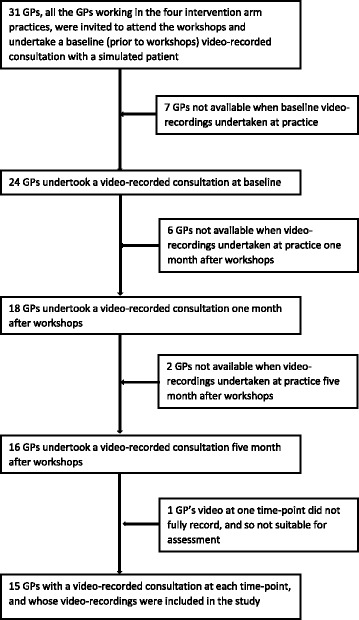
Participant recruitment flowchart

Thirty-one GPs in total were eligible to take part from the practices participating in the MOSAICS trial. All were invited to attend the workshops and be video-recorded. Baseline videos were obtained for 24 GPs. Of these, there were 15 GPs who had a video suitable for analysis from all three time-points (baseline plus two follow-ups), resulting in a total of 45 videos assessed and used for the main analysis (full details given in Fig. 1 (participant recruitment flowchart)). Of the 15 GPs, 12 had attended all the workshops, two GPs two of the three workshops and one GP none.

Mean duration of all videos was 14.46 min (range of 8.80 to 26.93 min) with no difference in duration at the three time-points (paired *t* test: baseline v one-month, *p* = 0.28: baseline v five-months, *p* = 0.63; one-month v five-months, *p* = 0.13).

GP competency score increased after the workshops from median of seven consultation tasks undertaken before the workshops to 11 at both one-month and five-months after (Wilcoxon signed-rank test: one-month v baseline, *p* = 0.001; five-months v baseline, *p* = 0.001), see Table 2 for full details.

**Table 2:** Median, interquartile range and range of GP competency scores by time-point

	Baseline	1 month after workshops	5 months after workshops
Median	7	11*	11*
Interquartile range	5–9	10–12	10–11
Range	5–11	8–14	7–13

*Wilcoxon signed-rank test: one-month v baseline, *p* = 0.001; five-months v baseline, *p* = 0.001

Task delivery score was high at baseline for two tasks (eliciting what the patient is trying for pain, and advising or prescribing analgesia) which were present in all baseline videos. The score for six other tasks increased after the workshops at one-month (McNemar test: one-month v baseline, *p* < 0.05), with the increase for three of these tasks sustained at five-months (McNemar test: five-month v baseline, *p* < 0.05), see Table 3 for full details.

**Table 3:** Task delivery score by consultation task by time-point and comparison of one- and five-months with baseline

Consultation task	Task delivery score			Change in task delivery scores, *p* values from McNemar test	
	Baseline	1 month after	5 months after	1 month v baseline	5 months v baseline
Eliciting ideas about problem	11	11	8	0.62	0.45
Giving the diagnosis of OA, using the word “osteoarthritis”	10	6	9	0.22	1.00
Eliciting understanding of OA	1	2	4	1.00	0.37
Explaining that OA does not get inevitably worse	4	13	14	0.01	0.01
Explaining that OA is treatable	9	15	14	0.04	0.13
Eliciting expectations of the consultation	6	14	11	0.01	0.13
Addressing expectations	6	13	11	0.02	0.13
Eliciting what the patient is trying for pain	15	15	14	-^a^	1.00
Advising or prescribing analgesia	15	14	15	1.00	-^a^
Eliciting what the patient is trying for the problem other analgesia	6	9	3	0.51	0.45
Advising that exercise is beneficial	12	15	15	0.25	0.25
Advising that weight loss is beneficial	10	6	11	0.13	1.00
Offering general written info	4	14	14	0.004	0.004
Offering a nurse appointment to help with the problem	0	15	15	0.000	0.000

^a^McNemar Test not computable

The task delivery score for “Giving the diagnosis of OA using the word “osteoarthritis”” decreased non-significantly after the workshops (Table 3), an unexpected finding given this task was a key focus of the workshops. Further analysis determined that the reason for the low task delivery scores after the workshops was that the diagnosis was often given using the word “arthritis”, and not “osteoarthritis” (see Additional file 4).

## Discussion

This study has demonstrated that GP competency for undertaking consultations for OA can be significantly increased by workshops. After the workshops, GPs were undertaking a median of 11 of 14 pre-determined OA consultation tasks in each consultation, and nine of the tasks were being undertaken by at least 80% of GPs at each consultation. Six tasks increased in frequency from baseline and were those relating to: giving more positive explanations about OA, eliciting and addressing patient expectations about the consultation, offering written information, and, in the context of the MOSAICS trial, offering a follow-up in a nurse-led OA clinic. Tasks relating to managing pain and advising on exercise were undertaken by nearly all GPs at baseline and continued to be so after the workshops, but those relating to eliciting patient ideas about their problem, their understanding of OA, and their prior use of non-pharmacological treatments were not increased by the workshops and were variably undertaken by the GPs.

Despite being a specific focus of the workshops, using the word “osteoarthritis” when giving the diagnosis of OA did not increase after the workshops, with OA often being referred to as “arthritis”. This finding aligns with the conclusion from a recent observational study on real-life consultations for OA that there is much confusion as to how to portray and explain OA in the general practice consultation [10]. The average duration of the video-recorded consultations was longer than GP consultations in day-to-day practice, and may reflect the effect of being observed, but the increase in competency was not associated with longer consultations. This is an important finding for generalisability and implementation of the optimal consultation.

To our knowledge this is the first study which has evaluated the effect of a behaviour change intervention to optimise GP consultations for OA on clinical practice. Other studies on primary care management of OA have evaluated the effect of standardised consultations [24], interactive peer-group training [25] and a training course about OA care [26] on patient outcomes but not clinical practice. Studies which have evaluated the effect of workshops using a similar approach to skills training for GPs as used in this study, those based on the “context-bound” skills training method [27] (a method which focuses on the management of the clinical problem and not simply the uptake of specific communication skills), have shown that this approach can significantly enhance clinical practice in consultations for other conditions [28, 29].

Study strengths included the robust assessment methodology used, with each assessor viewing all the videos of an individual GP in random order and blinded to time-point, and the establishment of validity and reliability of use of the video rating tool. All bar one of the GPs included in the evaluation had attended two or more workshops.

Study limitations included the use of a non-randomised before and after design to evaluate the workshops, but the logistics of embedding the study in the intervention arm practices of the MOSAICS trial precluded randomising GPs to training or not training, as all needed to be trained to deliver the trial intervention. In addition resources precluded undertaking video-recorded consultations with control arm practices. However, the use of paired before and after observations enabled us to control for the effect of GP characteristics differing between time-points. A possible limitation is that in two practices not all the GPs invited to attend the training were included in this study as not all had a full set of video-consultations. This could have resulted in selection bias in that those included might not have been typical of all GPs from these practices. The GPs who were included might have been more committed to the study, in that they undertook all the videos, and so may have been more motivated to enhance their clinical practice. However, two of the 11 GPs included from these practices were only “partially trained” and one not trained at all, suggesting that even among the included GPs there was a range of commitment to the study. Furthermore, whether GPs had a full set of videos for assessment, or not, was due to whether they were working in the practice on the days the videos were undertaken and not solely to their commitment to the study: the research nurse endeavoured to video all GPs who were present in the surgery at the time of the video sessions. Another possible limitation, with the use of repeat video-recorded consultations to evaluate the workshops, is that of a learning effect from previously undertaking the consultation. We addressed this by using different simulated patients with different scenarios at each of the three time-points.

The finding that the workshops increased GP competency for OA consultations should be generalisable to GPs as a whole: although the GPs in this study were those in practices which had signed up to participating in the MOSAICS trial, and so may have differed from GPs as a whole, their reported views and practice at baseline on OA management were similar to those reported by GPs more generally [30, 31]. Although the findings are for GP competency for OA consultations, and not performance, the consultations with simulated patients were made as real and naturalistic as possible by undertaking them in the GPs’ own surgeries and by having detailed and realistic patient scenarios and biographies (see Additional file 2). The use of the workshops, in the specific manner in which they were delivered in this study, to enhance other GPs’ clinical practice for OA may not be feasible across the health service as a whole: the workshops were quite labour-intensive to deliver and required GPs to commit considerable time to attendance. In the UK NICE has recently updated its guidance on the care of people with OA [32] and, given current evidence on the management of OA in general practice in the UK, implementation of its recommendations will require GP consultations for OA to be optimised. The evidence from this study, on how to enhance GP competency for such consultations, will contribute to developing training resources for more general use, evidenced by its use in the development of written [33] and on-line [34] educational material for UK GPs and in underpinning OA implementation projects in UK regionally and in Europe. One issue that the detailed information from the current study will enable us to reflect on and consider is whether the content and delivery of training can be streamlined and made more efficient for general use without reducing its effectiveness in changing GP behaviour.

Further research is needed on how to help GPs better communicate with patients about OA, both in terms of what to call it, which was not resolved by this study, and of how to explain what it is. The latter can build on the approach taken in this study which led to GPs giving more positive OA explanations, namely that OA is treatable and is not inevitably progressive.

## Conclusions

In summary, this before and after study has demonstrated that GP competency in undertaking consultations for OA can be enhanced. The workshops evaluated in this study to enhance competence can inform development of future generalised initiatives to enhance OA care in general practice.

## Additional files

Additional file 1: GREET 2015 checklist for the behaviour change intervention workshops. (DOCX 25 kb)

Additional file 2: Development of simulated patient scenarios and biographies (a description of their development and a synopsis of the six scenarios developed), and arrangements for undertaking video-recorded simulated patient consultations. (DOCX 22 kb)

Additional file 3: Validity, and reliability in use, of the video rating tool - methodology and results of establishing validity and reliability of the video rating tool. (DOCX 93 kb)

Additional file 4: Further analysis of task delivery score for “Giving the diagnosis of OA using the word “osteoarthritis – methodology” and results of further analysis undertaken”. (DOCX 15 kb)

## Abbreviations

BCI: Behaviour change intervention
GP: General practitioner
MOSAICS trial: Management of OsteoArthritis in Consultations trial
NICE: National Institute for Health and Care Excellence
OA: Osteoarthritis
UK: United Kingdom
