# Supplementary material for: Enhancing delivery of osteoarthritis care in the general practice consultation: evaluation of a behaviour change intervention
Source: BMC Fam Pract. 2018 Feb 6;19:26. doi: 10.1186/s12875-018-0715-8 (PMC5801670; doi:10.1186/s12875-018-0715-8)
Supplement: Supplementary file 1 — GREET 2015 checklist for the behaviour change intervention workshops. (DOCX 25 kb) [file 12875_2018_715_MOESM1_ESM.docx]

| **Additional file 1: GREET 2015 checklist for the behaviour change intervention workshops** |  |
| --- | --- |
| BRIEF NAME | |
| 1. INTERVENTION: The intervention consisted of a series of four workshops to enhance GP clinical behaviour for patients presenting with possible osteoarthritis (OA), and addressed determinants of behaviour change previously identified as relevant to this behaviour [1]. | |
| WHY - this educational process | |
| 2. THEORY: The intervention was based on an implementation of change model and utilized the Theoretical Domains Framework and Adult Learning Theory [1]. | |
| 3. LEARNING OBJECTIVES: By the end of this intervention the GPs were expected to: have the necessary knowledge and skills to diagnosis and initially manage patients presenting with peripheral joint pain in line with NICE OA guidance [2]; helpful beliefs about undertaking this activity; be motivated, and remember, to undertake this activity in day-to-day practice. | |
| 4. EBP CONTENT: none – the intervention was not an EBP intervention. | |
| WHAT | |
| 5. MATERIALS: GPs were provided with a workshop handbook containing: the Keele Guide for People who have Osteoarthritis [3], the NICE OA 2008 Quick Reference Guide (no longer available on-line), the Arthritis Research UK Hands On leaflet on OA [4], (and other written information on the management of OA), copies of presentations given in the workshops, and an aide-memoire for an enhanced GP OA consultation. Simulated patients were utilized in skills training sessions. | |
| 6. EDUCATIONAL STRATEGIES: The workshops used a mixture of didactic and interactive sessions, with specific skills training sessions based on the ‘context-bound communication skills training’ approach [5], including the use of video recorded GP / simulated patient OA consultations undertaken during day-to-day practice. | |
| 7. INCENTIVES: Practices were reimbursed for GP time: to attend the workshops, and to deliver the enhanced OA consultation in day-to-day practice. | |
| WHO PROVIDED | |
| 8. INSTRUCTORS: The workshops were delivered by a team of academic clinicians: a GP with an interest in OA, a GP experienced at running skills training sessions, a physiotherapist specializing in musculoskeletal problems, a clinical psychologist with expertise in the management of chronic pain, and a rheumatologist with an interest in OA. | |
| HOW | |
| 9. DELIVERY: The workshops were delivered in group face-to-face sessions with a learner to instructor ratio varying between 1 to 1 and 2 to 1. | |
| HERE | |
| 10. ENVIRONMENT: The majority of the workshops were delivered at the practices in a convenient meeting room, three were delivered in a university facility (which was mutually convenient for GPs from two practices). | |
| WHEN and HOW MUCH | |
| 11. SCHEDULE: The four workshops were delivered as a series to each practice at approximately two-weekly intervals with the first three lasting two hours each, and the last an hour. | |
| 12. TIME UNDERTAKEN BY LEARNERS: The GPs spent in total seven hours in face-to-face contact with instructors, were asked to read the Keele OA Guide [3] between workshops 1 and 2 (taking approximately 30 minutes), and undertook consultations at their practices with simulated patients lasting in total about one hour. | |
| PLANNED CHANGES | |
| 13. LEARNER SPECIFIC ADAPTION: The intervention was tailor-made for the study but was not adapted for GPs in a specific practice other than that required for the workshops to address the learning needs of individual GPs. | |
| UNPLANNED CHANGES | |
| 14. MODIFICATION DURING DELIVERY: No unplanned changes were made. | |
| HOW WELL | |
| 15. ATTENDANCE: Workshop attendance was assessed by the use of attendance registers. The table show the attendance by practice.   \|  \| **Number (%) GPs who attended workshop** \| \| \| \| \| --- \| --- \| --- \| --- \| --- \| \|  \| **Practice A (n=2)*** \| **Practice B (n=7)*** \| **Practice C (n=20)*** \| **Practice D (n=2)*** \| \| **Workshop 1** \| 2 (100) \| 5 (71) \| 13 (65) \| 2 (100) \| \| **Workshop 2** \| 2 (100) \| 6 (86) \| 14 (70) \| 2 (100) \| \| **Workshop 3** \| 2 (100) \| 5 (71) \| 15 (75) \| 2 (100) \| \| **Workshop 4** \| 2 (100) \| 3 (43) \| 6 (30) \| 2 (100) \|   Workshop attendance by practice.  * number of GPs working in the practice and invited to attend the workshops  Attendance was facilitated by arranging the workshops at times convenient to the GPs of an individual practice, and, in the majority of cases, at their practices | |
| 16. ASSESSMENY OF DELIVERY: A detailed log and field notes were made by the team delivering the workshops and were used to undertake an audit of workshop delivery. | |
| 17. DELIVERY AS PLANNED: This was assessed as part of the workshop delivery audit and all four workshops to all four practices were delivered as planned. | |
| REFERENCES  1 Porcheret M, Main C, Croft P, McKinley R, Hassell A, Dziedzic K. Development of a behaviour change intervention: a case study on the practical application of theory. Implement Sci 2014;9(1):42,5908-9-42. | |
| 2 Conaghan PG, Dickson J, Grant RL, Guideline DG. Care and management of osteoarthritis in adults: summary of NICE guidance*. BMJ* 2008;336(7642):502-3  3 Guide for People who have Osteoarthritis. Keele University. Available at: <https://www.arthritisresearchuk.org/arthritis-information/keele-oa-guide.aspx> Accessed 05/10/2017  4 Osteoarthritis: a modern approach to diagnosis and management. Available at: [http://www.arthritisresearchuk.org/health-professionals-and-students/reports/hands-on/hands-on-autumn-2011.aspx Accessed 05/10/2017](http://www.arthritisresearchuk.org/health-professionals-and-students/reports/hands-on/hands-on-autumn-2011.aspx%20Accessed%2005/10/2017)  5 Rollnick S, Kinnersley P, Butler C. Context-bound communication skills training: development of a new method. Med Educ 2002;36(4):377-83. | |
