# Supplementary material for: Enhancing delivery of osteoarthritis care in the general practice consultation: evaluation of a behaviour change intervention
Source: BMC Fam Pract. 2018 Feb 6;19:26. doi: 10.1186/s12875-018-0715-8 (PMC5801670; doi:10.1186/s12875-018-0715-8)
Supplement: Supplementary file 2 — Development of simulated patient scenarios and biographies (a description of their development and a synopsis of the six scenarios developed), and arrangements for undertaking video-recorded simulated patient consultations. (DOCX 22 kb) [file 12875_2018_715_MOESM2_ESM.docx]

**ADDITIONAL FILE 2**

**Development of simulated patient scenarios and biographies, and arrangements for undertaking video-recorded simulated patient consultations**

The simulated patient scenarios and biographies were developed in five stages:

1. Construction of the scenario framework: i) presenting problem, ii) past medical and social history, iii) ideas and concerns about the problem, expectations about the consultation and iv) knowledge and beliefs about OA and its treatment
2. Listing of issues for inclusion relevant to delivery of key elements of the “enhanced OA consultation”: i) what OA is, its prognosis and treatment, ii) pain management for OA, and iii) self-management of OA, iv) exercise and physical activity for OA and v) diet and weight loss
3. Identification of patient ideas, beliefs, attitudes, expectations about issues listed at stage 2, which had been reported in the literature
4. Development of three basic patient scenarios, each one representing different ideas and concerns about the nature of OA and different co-morbidities, and each one covering a different aspect of self-management: exercise, weight loss and pain management. Each basic scenario had two versions: in one the simulated patient presented with chronic knee pain and in the other with chronic hip pain. Synopses of the six scenarios are shown in additional table 5
5. Each scenario was allocated to a simulator (a person who would be the simulated patient) who developed the biography for the simulated patient with that scenario which they could realistically portray

Additional table 5 Synopses of six scenarios developed by issues addressed by scenario

| **Scenario synopsis** | **Issues addressed** |
| --- | --- |
| Scenarios A (knee) and B (hip) consisted of a patient with ischaemic heart disease, who had tried simple analgesia and thought their problem was due to “wear and tear”. They had concerns about exercise for example, that exercise was not safe and that it was difficult to exercise locally (appendix 5.11 page 372) | Idea that the problem was due to “wear and tear”  Worried about exercise: not safe and lack of access |
| Scenarios C (knee) and D (hip) consisted of a patient with diabetes who had tried over the counter painkillers and was concerned they had rheumatoid arthritis. They were overweight and had tried to lose weight many times before and had not succeeded (appendix 5.12 page 374) | Concern that the problem was due to rheumatoid arthritis  Negative previous experience of losing weight |
| Scenarios E (knee) and F (hip) consisted of a patient with hypothyroidism who only occasionally took painkillers and thought they had arthritis as they were getting older. They had concerns about taking tablets which they thought were addictive and often give them side effects (appendix 5.13 page 376) | Idea that the problem was due to “arthritis” due to ageing  Worried about taking analgesia: addictive and frequent side effects |

Arrangements for undertaking video-recorded simulated patient consultations

To undertake the video-recorded consultations four simulators were recruited, and each presented to the GPs with one of the six scenarios we developed (see above). We were careful to make sure that at each time point the GPs had not seen the simulated patient, nor the scenario they portrayed, previously (additional table 6)

Additional table 5 Simulated patient (initials of simulator shown) and scenario portrayed, by practice and time-point of video-recorded consultation

| Time-point of video | Practice A | Practice B | Practice C | Practice D |
| --- | --- | --- | --- | --- |
| Baseline | SM with scenario D | SM with scenario D | SM with scenario D or JC with scenario F* | SM with scenario D |
| One month after workshops | JC with scenario F | JC with scenario F | DM with scenario A | DM with scenario A |
| Five months after workshops | BM with scenario E | BM with scenario E | BM with scenario E | BM with scenario E |

* Two video sessions were needed with different simulators available
