# Supplementary material for: Enhancing delivery of osteoarthritis care in the general practice consultation: evaluation of a behaviour change intervention
Source: BMC Fam Pract. 2018 Feb 6;19:26. doi: 10.1186/s12875-018-0715-8 (PMC5801670; doi:10.1186/s12875-018-0715-8)
Supplement: Supplementary file 3 — Validity, and reliability in use, of the video rating tool - methodology and results of establishing validity and reliability of the video rating tool. (DOCX 93 kb) [file 12875_2018_715_MOESM3_ESM.docx]

**Additional file 3**

**Assessor Training**

Four GPs, not previously involved with or participating in the MOSAICS study, were recruited as assessors to rate the videos, and attended a two-hour training session led by MP. The MOSAICS trial, details of the workshops, and methodology for undertaking the videos were briefly presented. The four GPs were taken through the use of the rating tool (see appendix 1 (recording sheet) and appendix 2 (instructions for use) in detail. They then independently rated two demonstration videos of the model OA consultation for which standard ratings had been developed.

Assessors’ ratings were compared with the standard ratings for the two demonstration videos, and the assessors received individual feedback on their ratings. The feedback focused on their assessment of tasks for which their rating differed from standard rating, and there was discussion about how they had applied the criteria compared with how the criteria had been applied in the standard rating. The aim was, through discussion, to achieve a consistent approach by the assessors to rating videos. Further feedback and training was undertaken after the criterion validity exercise (see below)

**Establishment of criterion validity of use of video rating tool**

A comparison was undertaken of assessors’ ratings of video-recorded consultations with standard ratings for the same videos.

Standard ratings were developed by an expert panel (MP, CM, PC, KD) for five randomly selected video-recorded consultations, stratified so that the five videos included: a video of a GP from each of the four intervention arm practices, videos undertaken before and after the training workshops, and for one GP a pair of videos (one before and one after the workshops). The members of the expert panel individually and independently rated each of videos, met to compare ratings, and agreed standard ratings by discussion.

Four assessors were trained in the use of the rating tool and following training rated the five videos for which there were standard ratings. For each assessor a two by two table was constructed of their rating of the 14 tasks in the five videos, 70 tasks in total, and compared with the standard ratings. Percentage agreement, sensitivity and specificity, compared with the standard scores, were calculated for each assessor (additional table 1). Their results were fed back to assessors, discrepancies discussed and further training given on the assessment of tasks, for which there had been demonstrable discrepancy, and about deciding when these were, or were not, present on the videos.

Additional table 1 Percentage agreement, sensitivity and specificity of assessor ratings compared with standard ratings

| **Measure** | **Assessor** | | | |
| --- | --- | --- | --- | --- |
|  | **1** | **2** | **3** | **4** |
| **Percentage agreement (%)** | 86 | 84 | 84 | 80 |
| **Sensitivity (%)** | 98 | 89 | 85 | 98 |
| **Specificity (%)** | 63 | 75 | 83 | 46 |

**Establishment of inter-observer reliability of use of the video rating tool**

An inter-observer ranking analysis was undertaken on the assessment of the five videos (rated in the validity exercise described above) to determine if the assessors ranked the videos in the same order as each other for GP competency score (see main text for definition of score) and how their rankings compared with those derived from the standard ratings (termed standard rankings).

GP competency scores for each assessor, and for the standard rating, of each video were calculated. A ranking of “1” was given to the video with the lowest GP competency score and so on up to a ranking of “5” for the video with the highest GP competency score. When the rankings of videos were tied, the mean rank was determined. Kendall’s coefficient of concordance was used to compare the rankings of the four GP assessors, a descriptive comparison was used to compare assessor rankings with rankings from the standard rankings.

The GP competency scores and the rankings of the five videos are shown in additional table 2. Kendall’s coefficient of concordance test applied to the rankings by the four assessors was 0.79 (p <0.01), indicating that there was good inter-observer reliability in ranking the videos by GP competency score.

Additional table 2 GP competency score (denoted GP score in table) and ranking of five videos by assessor

| **Video** | **Assessor 1** | | **Assessor 2** | | **Assessor 3** | | **Assessor 4** | |
| --- | --- | --- | --- | --- | --- | --- | --- | --- |
|  | **GP score** | **Ranking** | **GP score** | **Ranking** | **GP score** | **Ranking** | **GP score** | **Ranking** |
| **A** | 11 | **3** | 9 | **3** | 10 | **1=** (2) | 8 | **2** |
| **B** | 13 | **5** | 9 | **3** | 14 | **4=** (4.5) | 9 | **3=** (3.5) |
| **C** | 9 | **1=** (1.5) | 9 | **3** | 10 | **1=** (2) | 9 | **3=** (3.5) |
| **D** | 9 | **1=** (1.5) | 7 | **1** | 10 | **1=** (2) | 6 | **1** |
| **E** | 12 | **4** | 13 | **5** | 14 | 4= (4.5) | 11 | **5** |

The comparison of the standard rankings with assessor rankings is shown in additional table 3. From the table it can be seen that the order in which the videos were ranked by the assessors closely matched the order from the standard rating. For assessors 2 and 3 the ranking is in sequence with the rating standard, and for assessors 1 and 4 the ranking of one video is out of sequence.

Additional table 3 Videos ordered by standard ranking, by assessor ranking

| **Video** | **Standard ranking** | **Assessor 1** | **Assessor 2** | **Assessor 3** | **Assessor 4** |
| --- | --- | --- | --- | --- | --- |
| **D** | 1 | 1= | 1 | 1= | 1 |
| **C** | 2 | 1= | 3= | 1= | 3= |
| **A** | 3 | 3 | 3= | 1= | 2 |
| **B** | 4 | 5 | 3= | 4= | 3= |
| **E** | 5 | 4 | 5 | 4= | 5 |

Appendix 1 – Video assessment recording sheet


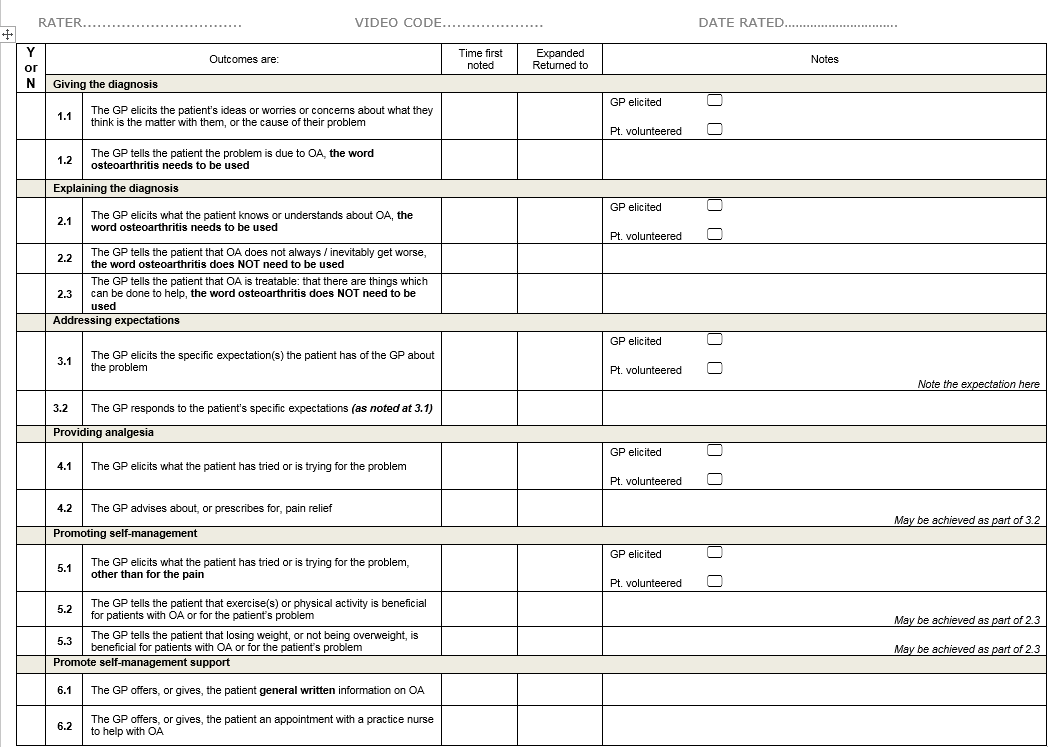


Appendix 2 – Instructions for assessing videos

1. Fill in your name, video code and date the rating was undertaken
2. Watch each video recording in the order listed on your sheet (O:\MOAC video rating)
3. When you see/hear one of the outcomes stop the video & note the time on the video when this was first seen/heard (you may need to rewind slightly to note the time)
4. If outcome “expanded on / returned to” tick in this column
5. Note very briefly, in the “notes” column, what you heard or saw for this outcome.
6. Points to note when rating

1. Opening GP questions such as “what can I do to help?”, or “how can I help today?” do not represent “eliciting expectations” – item 3.1
2. Items 1.1, 2.1, 3.1, 4.1 and 5.1 are preludes to the provision of information and need to be scored in reference to this – enough information needs to have been elicited for the provision of information to be tailored to the patient

1.1 so that the diagnosis is given in light of what the patient thinks the problem is

2.1 so that the OA explanation is tailored to the patient’s prior knowledge / understanding

3.1 so that the patient’s **specific** expectation can be addressed

4.1 and 5.1 so that advice on pain and self-management is tailored to what the patient has tried / is trying

1. Items above may be GP elicited or patient volunteered, or both, please note
2. For item 3.1 please note the patient’s expectation in the “notes” column
3. Items 5.2 and/or 5.3 may be achieved as part of 2.3 – score **all** if achieved
4. Item 4.2 may be achieved as part of 3.2 (if pain relief was the specific expectation) – score **both** if achieved
5. 6.1 is only achieved if **genera**l **written** information about OA is given, and not specific info on one aspect of care. This is the first appointment for the problem and the patient needs to be given some **general written** info on OA
6. Item 6.2 is only achieved if the nurse appointment is for the general management of OA, and not one specific issue such as weight loss
7. If you need to check the rating please feel free to review any part of the video – you do not need to look at the video just once
8. When you are satisfied with your rating fill in in the first column for your overall assessment: outcome achieved yes or no
9. Then put the completed form in the envelop provided and do not re-rate it in light of watching subsequent videos
10. When you have completed all the videos allocated to you please seal the envelop and return it to MP
