# Supplementary material for: Enhancing delivery of osteoarthritis care in the general practice consultation: evaluation of a behaviour change intervention
Source: BMC Fam Pract. 2018 Feb 6;19:26. doi: 10.1186/s12875-018-0715-8 (PMC5801670; doi:10.1186/s12875-018-0715-8)
Supplement: Supplementary file 4 — Further analysis of task delivery score for “Giving the diagnosis of OA using the word “osteoarthritis – methodology” and results of further analysis undertaken”. (DOCX 15 kb) [file 12875_2018_715_MOESM4_ESM.docx]

**ADDITIONAL FILE 4**

**Further analysis of task delivery score for “Giving the diagnosis of OA using the word “osteoarthritis””**

A key focus of the skills training sessions in the workshops was use of the word “osteoarthritis” when giving the diagnosis: to distinguish OA from rheumatoid arthritis and as a prelude to offering a guidebook entitled “A guide for people who have osteoarthritis”. The finding that fewer GPs, though not significantly fewer, had undertaken the task of giving the diagnosis of OA using the word “osteoarthritis” at 1 month after workshops compared with baseline was unexpected. An analysis of the individual GP change in the delivery of this task was undertaken (additional table 4). In summary, five GPs delivered the task in all their videos, and four did not deliver it any video. One GP started to deliver it after workshops and two GPs stopped after workshops. And three GPs did not deliver it at 1 month, but did at baseline and 5 months.

Additional table 4 Individual GP delivery of giving the diagnosis using the word "osteoarthritis" by time-point

| **GP** | **Presence (1) or absence (0) of task** | | |
| --- | --- | --- | --- |
|  | **Baseline** | **1 month after**  **workshops** | **5 months after**  **workshops** |
| **41** | 1 | 0 | 1 |
| **24** | 1 | 1 | 1 |
| **34** | 0 | 0 | 0 |
| **45** | 1 | 1 | 1 |
| **33** | 0 | 0 | 0 |
| **37** | 1 | 1 | 1 |
| **40** | 1 | 1 | 1 |
| **28** | 0 | 0 | 0 |
| **10** | 0 | 1 | 1 |
| **35** | 1 | 0 | 1 |
| **31** | 1 | 0 | 0 |
| **29** | 1 | 0 | 1 |
| **48** | 1 | 1 | 1 |
| **26** | 1 | 0 | 0 |
| **44** | 0 | 0 | 0 |

Further assessment was undertaken to investigate whether non-delivery of the task was a consequence of: i) the GP not giving the diagnosis in the video or ii) the GP giving the diagnosis but using an alternative word or phrase to osteoarthritis. Non-delivery of the task was found in 20 of the 45 videos (additional table 4). These 20 videos were viewed and an assessment was made on: i) whether the diagnosis was given and ii) if so what words or phrases were actually used. In 16 of the 20 videos the diagnosis was assessed as given and in 15 of these the word “arthritis” was used to give the diagnosis. Other additional words or phrases used were “wear and tear” and “degeneration” (additional table 5).

Additional table 5 Further assessment of the 20 videos in which the tasks of “giving the diagnosis with the use of the word “osteoarthritis”” not delivered

| **GP** | **Diagnosis given or not given and if given (words / phrases used)** | | |
| --- | --- | --- | --- |
|  | **Baseline** | **1 month after**  **workshops** | **5 months after**  **workshops** |
| 41 |  | Given  (arthritis) |  |
| 34 | Given  (arthritis) | Given  (arthritis) | Given  (arthritis) |
| 33 | Given  (wear and tear, arthritis) | Given  (arthritis) | Given  (arthritis, wear and tear arthritis) |
| 28 | Given  (arthritis) | Given  (arthritis, arthritic hip) | Given  (wear and tear, degeneration, arthritis, arthritic joint) |
| 10 | Given  (arthritis) |  |  |
| 35 |  | Given  (arthritis) |  |
| 31 |  | Not given | Given  (arthritis) |
| 29 |  | Given  (arthritis) |  |
| 26 |  | Not given | Given  (arthritis) |
| 44 | Not given | Not given | Given  (wear and tear) |
